# Preliminary Assessment of Microalgae, *Chlorella vulgaris*, and Black Soldier Fly, *Hermetia illucens* Larvae, as a Viable Alternative Feed Combination for Red Hybrid Tilapia, *Oreochromis* sp. Culture

**DOI:** 10.1155/anu/5520954

**Published:** 2025-09-24

**Authors:** Aslah Mohamad, Ina Salwany Md Yasin, Mohamad Azzam-Sayuti, Muhammad Farhan Nazarudin, Amir-Danial Zahaludin, Sani Bashir, Azfar Ismail, Alan Tan Chee Yong, C. T. Tong

**Affiliations:** ^1^Laboratory of Aquatic Animal Health and Therapeutics, Institute of Bioscience, Universiti Putra Malaysia, UPM Serdang 43400, Selangor, Malaysia; ^2^China-Asean College of Marine Sciences, Xiamen University Malaysia, Jalan Sunsuria, Bandar Sunsuria, Sepang 43900, Selangor, Malaysia; ^3^Department of Aquaculture, Faculty of Agriculture, Universiti Putra Malaysia, UPM Serdang 43400, Selangor, Malaysia; ^4^Department of Veterinary Laboratory Diagnostic, Faculty of Veterinary Medicine, Universiti Putra Malaysia, UPM Serdang 43400, Selangor, Malaysia; ^5^World New Energy Centre Sdn Bhd. GM3-3, 3rd Floor, GMBB Kompleks, 2, Jalan Robertson, Bukit Bintang 50150, Kuala Lumpur, Wilayah Persekutuan, Malaysia

**Keywords:** black soldier fly, *Chlorella vulgaris*, feeding trial, growth performance, microalgae

## Abstract

This study evaluates the potential of microalgae, *Chlorella vulgaris*, and black soldier fly larvae (BSFL), *Hermetia illucens*, as an alternative complete diet for red hybrid tilapia, addressing challenges arising from declining fish stocks and unsustainable aquaculture practices reliant on fish meal in commercial pellets. The study involved 270 tilapia separated into three groups: Group 1 received *C. vulgaris* alone, Group 2 was fed with a combination of BSFL and *C. vulgaris*, and Group 3 with commercial pellets (control) for 70 days. The results demonstrated that the combination of BSFL and *C. vulgaris* had a high potential to be an alternative to commercial pellets, with comparable growth performance with the control group. In contrast, fish-fed with *C. vulgaris* alone exhibited significantly slower growth rates and higher mortality. Economic analysis showed that the BSFL and *C. vulgaris* combination reduced feed costs by 59.40% and increased the profit index by 144.79% compared to the control group. However, the combination diet did not provide significant protection against streptococcosis compared to commercial pellets. This study highlights the potential of incorporating BSFL and *C. vulgaris* into tilapia diets to enhance sustainability and economic outcomes for farmers. It also emphasizes the role of alternative feeds in promoting environmentally sustainable aquaculture practices, with the goal of achieving zero-carbon emissions. This study is the earliest report on the direct combination of BSFL and *C. vulgaris* diet in tilapia, a globally cultivated aquaculture fish species.

## 1. Introduction

Aquaculture, a rapidly expanding sector in the food industry, has gained prominence due to the increasing global population and the need to meet the growing demand for food [1]. Fish has become a staple in human diets, providing protein, essential nutrients, and fats [2]. To reduce reliance on wild-caught fisheries, fish farming has become more prevalent. However, the increasing demand for fish has led to a corresponding increase in the need for fish feed. Moreover, challenges such as disease outbreaks and climate change have further exacerbated the decline in fish harvest. Microalgae have emerged as a potential alternative to traditional fish feed ingredients [3].

Conventional fish feed often includes ingredients like soybean meal and fish oil, contributing to deforestation and ecological disruption [4]. This reliance on unsustainable practices can lead to climate change issues, ultimately affecting the aquaculture sector [2]. In contrast, microalgae present several advantages, including higher areal biomass productivity compared to other photosynthetic organisms, while requiring substantially less water and land [5, 6]. This makes microalgae a highly promising option for sustainable fish feed. Additionally, microalgae are rich in essential amino acids, healthy triglycerides, vitamins, and other bioactive compounds that benefit fish health [4]. Previous studies have demonstrated that supplementation of microalgae in fish diets resulted in higher weight gain (WG) and improved feed conversion ratios (FCRs) compared to diets lacking microalgae [4]. Considering these factors, microalgae undoubtedly hold potential as substitutes for soybean meal and fish oil in fish feed formulations.

Alongside microalgae, insect meal is another promising alternative that is gaining attention in fish feed research. Insects offer unique nutritional benefits, including high levels of crude protein (CP), crude fat, and antimicrobial properties [7, 8]. The presence of chitin in insects serves as a prebiotic for the gastrointestinal tract (GIT) microbiome in fish, enhancing their immunity. Among the various insect species, black soldier flies (*Hermetia illucens*) have been extensively studied due to their rearing characteristics, such as short rearing periods, efficient conversion of waste to high-quality protein, and beneficial fatty acid composition [9]. Insect biomass can be efficiently processed through conventional methods like roasting, frying, and sun-drying or advanced techniques such as freeze-drying, oven-drying, and microwave-assisted drying.

In Malaysia, red hybrid tilapia (*Oreochromis* sp.) and Nile tilapia (*Oreochromis niloticus*) contribute substantially to freshwater aquaculture production [10]. Tilapia farming is widely favored due to its adaptability to diverse environmental settings, resilience to diseases, strong market demand, and ability to reach market size on various feed sources, including natural organisms and formulated feed [11]. In this study, we evaluated the effects of a combined diet incorporating microalgae and black soldier fly larvae (BSFL) as an alternative feed for tilapia. This approach aims to advance the development of sustainable and environmentally friendly aquaculture practices.

## 2. Materials and Methods

### 2.1. Culture Preparation and Diet

Microalgae, *Chlorella vulgaris* biomass and dried BSFL were supplied by the World New Energy Centre Sdn Bhd. Briefly, the *C. vulgaris* microalgae were provided in frozen block form, having been cultivated in Conway medium under controlled conditions, harvested through a flocculation process, and stored at −20°C. Prior to use, the frozen microalgae biomass was thawed and subsequently oven-dried at 40°C for 24 h. Meanwhile, for BSFL, the larvae were supplied in dried form, having been reared on domestic waste. The full-fat larvae were oven-dried at 70°C for 24 h before ground into a fine powder using a mechanical grinder. The BSFL powder was then stored at room temperature until further use.

For the experiment, three groups of experimental diets were arranged as follows: Group 1: microalgae, *Chlorella vulgaris* only, Group 2: a 1:1 mixture of *C. vulgaris* and BSFL powder, and Group 3: control group using commercial pellets (Star Feed, Klang, Selangor, Malaysia). For the Group 2 diet, tapioca starch powder was used as a binder, and water was added at 10% of the total feed weight to facilitate mixing and pellet formation using a 1 mm-diameter pellet extruder. The resulting pellets were then dried at 40°C for 24 h and stored at room temperature until further use.

### 2.2. Proximate Analysis

The biochemical composition of the feed used in this study was analyzed through a comprehensive proximate analysis focusing on seven key parameters: protein, moisture, ash, lipid, fiber, carbohydrate, and energy. The analyses were run by UNIPEQ Sdn. Bhd., Universiti Kebangsaan Malaysia, Malaysia, following the standardized methods outlined by the Association of Official Analytical Chemists (AOAC, 20^th^ ed.). Briefly, moisture content was measured by oven drying at 105°C until a constant weight was achieved, while ash content was determined through incineration in a muffle furnace at 550°C for 24 h. CP content (calculated as nitrogen × 6.25) and lipid content were assessed using the Kjeldahl and Soxhlet methods, respectively. Fiber content was determined using the Fibertec system, employing the Fibertec Hot Extraction Unit with 1.25% H_2_SO_4_ and NaOH. Carbohydrate content was estimated by difference, subtracting the measured values of moisture, protein, fat, and ash from the total. Energy content was measured using bomb calorimetry.

### 2.3. Experimental Fish

A total of 290 red hybrid tilapia (*Oreochromis* sp.) fingerlings (6.43 ± 0.15 g) were sourced from a commercial fish farm in Balakong, Selangor, and transported to the Laboratory of Aquatic Animals and Therapeutics (AquaHealth) at the Institute of Bioscience, Universiti Putra Malaysia (UPM). The fish were housed in 300–500 L indoor fiberglass tanks equipped with an aerated recirculating water system. Water quality parameters were maintained at 26.23 ± 0.59°C (temperature), 6.48 ± 0.24 (pH), 6.38 ± 0.48 mg/L (dissolved oxygen [DO]), and 0.01 ± 0.00 mg/L (ammonia; NH_3_).

Before the experiment, the fish underwent a 2-week acclimatization period under laboratory conditions. To assess health status, 20 fish were randomly selected for bacterial and parasitic screening, and no clinical signs of infection were observed. Water exchange was conducted twice weekly, replacing 20%–30% of the total volume.

### 2.4. Feeding Trial and Data Collection

**Figure 1:**
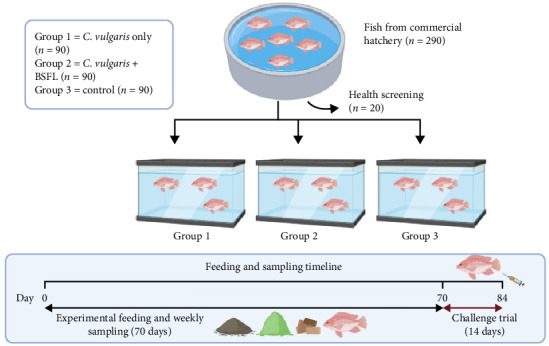
Experimental design of the study. The fish were assigned to three dietary treatments: a control diet, a microalgae-only diet, and a diet combining microalgae + black soldier fly larvae (*Hermetia illucens*). Each treatment was replicated in three tanks, and the feeding trial lasted 70 days. At the end of the trial, the fish were intraperitoneally injected with *Streptococcus agalactiae* at a concentration of 10^6^ CFU/L and observed for 14 days.

The experimental design is illustrated in Figure 1. Before the trial began, the fish were fasted for 24 h to standardize gut contents, then individually weighed and randomly assigned to nine rectangular aquarium tanks (122 cm × 46 cm × 46 cm), each containing 200 L of water. The stocking density was maintained at 0.15 fish per liter, with 30 fish per tank.

The feeding trial lasted 70 days, with fish assigned to one of three dietary treatments, each replicated in three tanks. Group 1 received microalgae biomass at 4% of their total body weight, directly added to the tanks. Group 2 was fed a combination of microalgae biomass (2% of body weight) and BSFL powder (2% of body weight). Group 3, serving as the control, was given commercial pellets (Star Feed, Klang, Selangor, Malaysia) at 4% of their total body weight. All groups were fed twice daily. The feeding amount was adjusted weekly based on updated weight measurements.

During the feeding trial period, 50% of the water was replaced with fresh dechlorinated water every 7 days with continuous aeration. The water quality parameters including temperature, turbidity, NH_3_, pH, DO, and ammonium (NH_4_^+^) were monitored twice per week throughout the 70-day feeding trial using a water quality analyzer (HYDROLAB HL7, HACH, Loveland, Colorado, USA) between 08:30 and 09:00 h.

Before stocking, each fish was individually weighed to calculate the mean initial weight (g) for each tank. Weekly sampling was conducted throughout the study period by randomly selecting 20 fish from each tank. The fish were weighed to monitor their growth over time, following the same procedures as at the start of the experiment.

### 2.5. Determination of Growth Performance

Every 7 days, 20 tilapia fingerlings from each treatment group were randomly weighed to determine their average body weight (g). The recorded weight data were used to evaluate growth performance based on the following parameters: total WG, specific growth rate (SGR), FCR, and feed efficiency (FE). These parameters were calculated using the formulas provided by Van Doan et al. [12]:

  $$\begin{array}{c} \text{Weight gain }\left(\text{WG; g}\right)=\text{Final body weight }-\text{initial body weight}, \end{array}$$

  $$\begin{array}{c} \text{Specific growth rate }\left(\text{SGR }\%\right)=100\times \left(\text{In final body weight}-\text{in initial body weight}\right)/\text{days},\; \end{array}$$

  $$\begin{array}{c} \text{Feed conversion ratio }\left(\text{FCR}\right)=\text{Feed intake}/\text{weight gain},\; \end{array}$$

  $$\begin{array}{c} \text{Feed efficiency }\left(\text{FE}\right)=\text{Weight gain of fish}/\text{feed consumed}, \end{array}$$

  $$\begin{array}{c} \text{Survival rate }\left(\%\right)=\left(\text{Final fish number}/\text{initial fish number}\right)\times 100\;. \end{array}$$

### 2.6. Bacterial Challenge

At the end of the 70-day feeding trial, the remaining fish were subjected to a bacterial challenge with *Streptococcus agalactiae*. The fish were intraperitoneally injected with live *S. agalactiae* at an LD_50_ concentration of 1.8 × 10^3^ CFU/mL [13]. Intraperitoneal (i.p.) injection was chosen as the infection route due to its efficiency in inducing disease symptoms within a shorter period.

The challenge was conducted in triplicate using 30 L glass tanks, with each tank containing 10 fish. Before the injection, fish were anesthetized with 105 mg/L of MS-222 (Sigma Aldrich, St. Louis, MO, USA). Postinfection, the fish were observed daily for 14 days to monitor clinical signs, abnormalities, and mortalities.

Survival percentage (%) was calculated using the following formula:

  $$\begin{array}{c} \text{Percentage of survival }\left(\%\right)=\left(\frac{\text{Final number of survived fish after }14-\text{day post challenge}}{\text{Initial number of fish before challenge}}\times 100\%\right). \end{array}$$

The moribund fish were subjected to bacterial isolation and PCR amplification using the specific primers F1 and IMOD [14], confirming the presence of *S. agalactiae* as evidenced by a specific band size of 220 bp.

### 2.7. Cost-Effectiveness Analysis

A partial enterprise budget was utilized to evaluate the cost-effectiveness of the microalgae and BSFL-based diets in this study. The diet cost was determined based on the market prices of the ingredients used (Table 1). The analysis was conducted using local market retail prices, converted to US dollars (USD 1 = RM 4.47) during the study period.

For the calculations, tank and labor costs were assumed to remain constant. The income generated from fish sales and the cost of feed were used to determine the incidence cost and profit index using the following formula [15]:

  $$\begin{array}{c} \text{Incidence cost }\left(\text{USD}\right)=\frac{\text{Amount of feed used }\left(\text{kg}\right)\times \text{price of feed per kg }\left(\text{USD}\right)}{\text{Total fish weight }\left(\text{kg}\right)}, \end{array}$$

  $$\begin{array}{c} \text{Profit index}=\frac{\text{Total fish weight }\left(\text{kg}\right)\times \text{ fish price per kg }\left(\text{USD}\right)\;}{\text{Amount of feed used }\left(\text{kg}\right)\times \text{ price of feed per kg }\left(\text{USD}\right)}. \end{array}$$

### 2.8. Statistical Analyses

Statistical analyses were conducted using IBM SPSS Statistics 26 (SPSS Inc., Chicago, IL, USA). A one-way ANOVA followed by Tukey's post hoc test was applied to determine significant differences among the treated and nontreated groups. Statistical significance was set at *p*  < 0.05. All results are presented as mean ± standard error (SE).

### 2.9. Ethical Statement

The authors confirm compliance with the journal's ethical policies as stated in the author guidelines. The study was conducted following the Institutional Animal Care and Use Committee (IACUC) guidelines of UPM and was approved under protocol number UPM/IACUC/AUP-R024/2024.

## 3. Results

### 3.1. Water Quality Parameters

**Figure 2:**
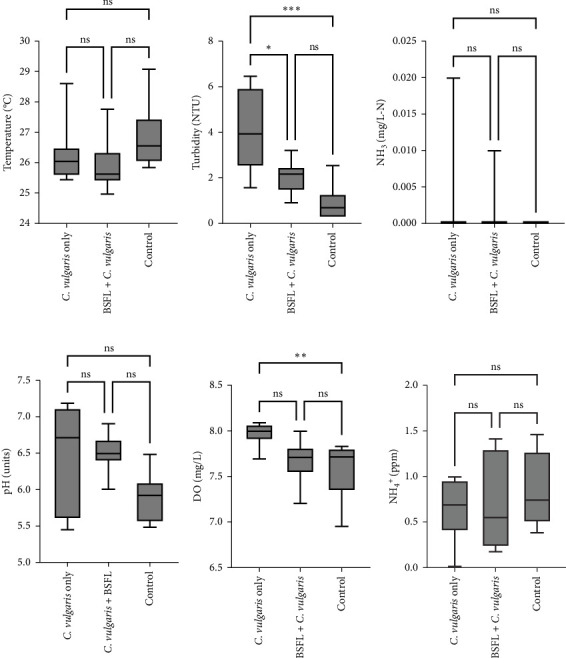
Water quality parameters measured in tanks used for rearing red hybrid tilapia-fed different diets: *C. vulgaris*-only, a combination of *C. vulgaris* and black soldier fly larvae (BSFL), and a control diet. The measured parameters include (A) water temperature, (B) turbidity, (C) ammonia (NH_3_), (D) pH, (E) dissolved oxygen (DO), and (F) ammonium (NH_4_^+^). Data are expressed as mean ± SD (*n* = 3). Box plots with different symbols (*⁣*^*∗*^, *⁣*^*∗∗*^, and *⁣*^*∗∗∗*^) indicate statistically significant differences at *p* < 0.05, *p* < 0.001, and *p* < 0.0001, respectively, while “ns” denotes no significant difference (*p* > 0.05).

The use of microalgae, *Chlorella vulgaris*, and BSFL as treatments significantly influenced some water quality parameters while others remained unaffected (Figure 2). The parameters measured include temperature, turbidity, NH_3_, pH, DO, and NH_4_^+^. Turbidity was significantly affected by the treatments (*p* < 0.0001). Water in tanks containing *C. vulgaris*-only had higher turbidity compared to both the *C. vulgaris* + BSFL treatment (*p* < 0.05) and the control group (*p* < 0.001). However, the turbidity in the *C. vulgaris* + BSFL tanks was not significantly different from the control (*p* > 0.05). DO was also significantly influenced by the treatments (*p* < 0.001). The *C. vulgaris* + BSFL treatment enhanced DO levels compared to the control group (*p* < 0.001). However, the DO levels in the *C. vulgaris*-only treatment were not significantly distinct from the *C. vulgaris* + BSFL or control groups (*p* > 0.05). On the other hand, temperature, pH, NH_3_, and NH_4_^+^ showed no significant differences (*p* > 0.05) across the treatments, indicating that the inclusion of *C. vulgaris* and BSFL did not alter these parameters, suggesting that these factors were stable under the experimental conditions.

### 3.2. Proximate Composition Analysis

The proximate composition analysis revealed notable differences in the nutritional profiles of the three feed types (*C. vulgaris*-only, *C. vulgaris* + BSFL, and a control feed) (Table 2). Moisture content was similar across the diets, ranging from 7.0 to 7.3 g/100 g. *C. vulgaris*-only diet had the highest protein content (54.7 g/100 g), while *C. vulgaris* + BSFL and the control commercial pellet had lower protein levels at 34.5 g/100 g and 32.2 g/100 g, respectively. The fat content was highest in *C. vulgaris* + BSFL (27.0 g/100 g), compared to minimal values in *C. vulgaris*-only diet (0.3 g/100 g) and the commercial pellet (4.3 g 100/g). Ash content was highest in the commercial pellet (9.6 g/100 g), moderate in *C. vulgaris* + BSFL (7.2 g/100 g), and lowest in *C. vulgaris*-only diet (5.1 g/100 g). Dietary fiber was highest in *C. vulgaris* + BSFL (21.9 g/100 g), followed by the commercial pellet (19.8 g/100 g), and lowest in *C. vulgaris*-only group (16.4 g/100 g). Carbohydrate content was highest in the commercial pellet (46.9 g/100 g), followed by *C. vulgaris*-only (32.7 g/100 g), and lowest in *C. vulgaris* + BSFL (25.0 g/100 g). Energy content was highest in *C. vulgaris* + BSFL (477 kcal/100 g) group, moderate in the commercial pellet (355 kcal/100 g), and lowest in *C. vulgaris*-only group (352 kcal/100 g).

### 3.3. Growth Performance

**Figure 3:**
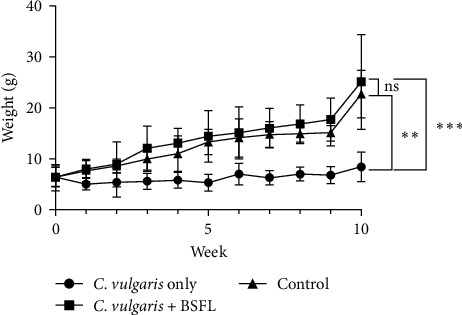
Fish growth curves for the 10-week study period for *C. vulgaris*-only, *C. vulgaris* + BSFL, and Control group. Curves with different symbols (*⁣*^*∗*^, *⁣*^*∗∗*^, and *⁣*^*∗∗∗*^) indicate statistically significant differences at *p* < 0.5, *p* < 0.001, and *p* < 0.0001 respectively, while “ns” denotes no significant difference (*p* > 0.05).

All treatment groups, except the *C. vulgaris*-only group, exhibited a positive growth trajectory during the 10-week feeding trial (Figure 3). The *C. vulgaris*-only group showed fluctuating and stagnant growth throughout the study. The BSFL + *C. vulgaris* group demonstrated a consistent increase in weight, achieving the highest final mean weight of 25.1 ± 9.32 g at Week 10. Similarly, the control group displayed a steady growth pattern but had a lower final mean weight of 22.7 ± 4.67 g by the end of the trial. In contrast, the *C. vulgaris*-only group exhibited minimal growth, with a significantly lower final mean weight of 8.4 ± 2.88 g compared to the other groups. Statistical analysis revealed significant differences in weight among the groups. The BSFL + *C. vulgaris* group showed significantly higher growth compared to the *C. vulgaris*-only group (*p* < 0.0001) and the control group (*p* < 0.001). However, no statistically significant difference (*p* > 0.05) was observed between the BSFL + *C. vulgaris* group and the control group during the earlier weeks until the end of the study.

WG rate (WGR) and SGR of the *C. vulgaris* + BSFL and control groups were comparable with no significant difference (*p* > 0.05) (Table 3). However, both groups exhibited significantly higher values than the *C. vulgaris*-only group (*p* < 0.05). The FCR was lowest in the *C. vulgaris* + BSFL group, followed by the control group, while the highest FCR was observed in the *C. vulgaris*-only group. Feed intake was significantly greater in the *C. vulgaris* + BSFL group, attributed to the higher body weight of the fish. FE in the *C. vulgaris* + BSFL group was not significant to the control group (*p* > 0.05) but significantly higher than the *C. vulgaris*-only group (*p* < 0.05). Survival was markedly lower in the *C. vulgaris*-only group, with a survival rate of 7.5% ± 3.54% over the 70-day feeding period. In contrast, the *C. vulgaris* + BSFL and control groups achieved survival rates of 90% ± 2.83% and 94.5% ± 0.71%, respectively, with no significant differences (*p* > 0.05).

### 3.4. Disease Resistance Against *Streptococcus agalactiae* in the Red Hybrid Tilapia

**Figure 4:**
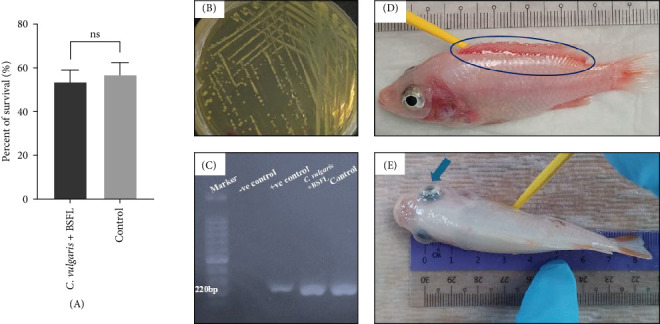
(A) Percentage of survival of *Oreochromis* sp. following challenge with *Streptococcus agalactiae*. No significant differences in survival were observed between the two dietary groups (*p* > 0.05). Data are presented as mean ± SE (*n* = 3). (B) Bacterial colonies grown on TSA agar, isolated from infected fish. (C) PCR amplification of *S. agalactiae* DNA using primers F1 and IMOD, confirming the presence of *S. agalactiae* with a specific band size of 220 bp in freshly deceased red hybrid tilapia. (D) Reddening of the dorsal fin (blue circle) in infected fish from the *C. vulgaris* + BSFL group. (E) Bilateral exophthalmia (blue arrow) was observed in infected fish from the Control group.

Mortality was monitored over 14 days following the bacterial challenge. The survival percentage of red hybrid tilapia exposed to a pathogenic strain of *S. agalactiae* in the experimental groups is shown in Figure 4. No significant differences in survival were observed between the *C. vulgaris* + BSFL group and the control group (*p* > 0.05). The *C. vulgaris*-only group was excluded from the challenge test due to the high mortality rate observed during the 70-day feeding trial. The presence of *S. agalactiae* in freshly deceased fish was confirmed through PCR amplification using primers F1 and IMOD, producing a specific band at 220 bp, as well as gross morphological signs observed in the infected fish.

### 3.5. Incidence Cost and Profit Index

The economic analysis in Table 4 compares the costs and profitability of red hybrid tilapia fed on three diets: *Chlorella vulgaris* only, *C. vulgaris* + BSFL, and a control diet. The parameters analyzed include the amount of feed used, feed cost, total fish weight, and profitability indicators over a 70-day feeding trial. In terms of feed input and cost, the *C. vulgaris* + BSFL diet required the highest amount of feed (3.87 kg/tank), followed by the control diet (3.51 kg/tank), while the *C. vulgaris-only* diet required the least (1.71 kg/tank). The price of the meal per kilogram was lowest for the *C. vulgaris*-only diet (0.45 USD/kg) and slightly higher for the *C. vulgaris* + BSFL diet (0.50 USD/kg). The control diet, however, was the most expensive at 1.17 USD/kg. Consequently, the feed price per tank followed a similar trend, with the *C. vulgaris-only* diet being the least costly (0.009 USD), the *C. vulgaris* + BSFL diet being moderately priced (0.022 USD), and the control diet being the most expensive (0.046 USD).

Economic metrics highlight the superior performance of the *C. vulgaris* + BSFL diet. The incidence cost, which represents the feed cost per kilogram of fish produced, was the lowest for this diet (0.95 USD), indicating high cost-effectiveness. In comparison, the control diet had a higher incidence cost (2.34 USD), and the *C. vulgaris*-only diet had the highest incidence cost (13.04 USD). The profit index further underscores the economic advantage of the *C. vulgaris* + BSFL diet, which achieved the highest value (2.35), indicating substantial profitability. The control diet yielded a moderate profit index (0.96), while the *C. vulgaris*-only diet performed poorly, with the lowest profit index (0.17).

## 4. Discussion

The growth performance results revealed that combining *C. vulgaris* and BSFL significantly enhanced growth metrics such as WG, SGR, and FCR compared to the *C. vulgaris*-only group. The comparable growth performance between the combination diet and the commercial pellets group is notable, indicating that the combination diet can be an alternative to commercial feeds without compromising productivity. These findings align with previous studies highlighting the nutritional richness of microalgae and insect larvae. For instance, *C. vulgaris* is recognized for its high protein content and essential nutrients, while BSFL provides high levels of fat, protein, and antimicrobial peptides beneficial for fish health and growth [4, 9]. Moreover, the inclusion of chitin from BSFL may have provided prebiotic effects, supporting gut health and nutrient absorption [8]. Several studies have similarly reported that the inclusion of microalgae [4, 5] and BSFL [16, 17] as a feed ingredient in fish diets contributes positively to growth performance, underscoring their potential as valuable feed ingredients in aquaculture.

The economic analysis demonstrated the significant cost-saving potential of the combination diet. A 59.40% reduction in incidence cost and a 144.79% improvement in profit index compared to the control diet highlight the financial advantages of integrating *C. vulgaris* and BSFL into tilapia feed. This reduction in feed cost is particularly impactful in the context of rising prices for traditional feed ingredients such as fishmeal and soybean meal, which are linked to unsustainable agricultural practices [5]. The minimal production costs of *C. vulgaris* and BSFL, coupled with their high nutritional value, make this combination diet a sustainable option for aquaculture. Additionally, the localized production of these feed components could reduce dependency on imported feed ingredients, further enhancing the economic resilience of aquaculture systems.

The combination diet aligns with the global emphasis on sustainable aquaculture. Microalgae cultivation is resource-efficient, requiring minimal land and water, and can utilize wastewater, thus contributing to a circular economy [6]. Similarly, BSFL can convert organic waste into high-quality protein, addressing waste management challenges while reducing the ecological footprint of feed production [9, 18].

Moreover, the stable water quality observed in the combined diet tanks was comparable to that of the control tanks with commercial pellets, further emphasizing the environmental compatibility of this diet. These findings suggest that the combination diet promotes fish growth and helps maintain optimal rearing conditions, thereby potentially reducing the environmental impacts associated with intensive aquaculture practices. The chemical and physical parameters of water quality closely influence the growth performance and survival rate of cultured fish. These parameters can be affected by fish metabolism, as nutrients absorbed from diets result in the production of fecal matter and urine, as well as the decomposition of uneaten feed [15]. Therefore, incorporating environmentally friendly ingredients that can replace fishmeal in aquafeeds, such as *C. vulgaris* and BSFL, without adversely affecting water quality is critical for achieving sustainable aquaculture.

Bioactive compounds in microalgae, such as carotenoids and polyunsaturated fatty acids, have been reported to enhance fish immune responses [4]. Abdel-Tawwab et al. [19] demonstrated that a *Chlorella*-formulated diet exhibited potent bactericidal activity against *S. agalactiae*, particularly at an inclusion level of 15 g/kg. Following bacterial challenge, fish-fed *Chlorella*-containing diets had a significantly higher survival rate (85%) than the control group (15%). This protective effect is attributed to *Chlorella*'s bioactive constituents, including carotenoids, chlorophyll, tocopherols, and polyphenols, which exert antioxidant activity by scavenging reactive oxygen species (ROS) and preventing lipid peroxidation [19].

Similarly, insect-derived chitin, such as that found in BSFL, has been shown to act as a prebiotic, modulating the gastrointestinal microbiome and enhancing immune function in fish [9]. Yildirim-Aksoy et al. [20] reported that diets containing 30% BSFL significantly improved survival rates following infection with *Flavobacterium columnare* and *Streptococcus iniae*, reaching 96.7% and 16.7%, respectively, compared to 81.7% and 7.3% in the control group. This improved survival was attributed to chitin-induced activation of the complement system via the alternative pathway, enhancing bacterial clearance by immune cells and promoting direct bacterial lysis through pore-forming complexes [20].

However, in this study, the combination diet did not improve disease resistance against *S. agalactiae*. This outcome may be attributed to an insufficient dosage or suboptimal ratio of microalgae and BSFL in the feed. This highlights the need for further optimization to enhance its immunomodulatory properties. Additionally, previous studies incorporated microalgae and BSFL as feed ingredients rather than as a direct feed, as used in this study [20]. The inclusion of other dietary components, such as vitamins, in those formulations may have contributed to improved disease resistance.

Besides, high mortality observed in the *C. vulgaris*-only group underscores the limitations of relying solely on microalgae as a complete feed. Although *Chlorella vulgaris*-based diets contain high protein levels, microalgae-derived proteins are limited by high fiber content, imbalanced amino acid profiles, and the presence of antinutritional factors such as phytic acid and protease inhibitors, which can impair nutrient absorption, reduce protein digestibility, and decrease feed intake [21, 22]. While tilapia, as omnivorous fish, are more tolerant of plant-based ingredients compared to carnivorous species, excessive inclusion of microalgae may still negatively affect growth and immune function. Studies have indicated that inclusion levels of microalgae exceeding 20% may result in nutrient imbalances, such as deficiencies in amino acids or minerals, within the test diets [23].Therefore, to meet the specific nutritional requirements of red hybrid tilapia, plant-based proteins must be carefully formulated and, when necessary, supplemented to ensure optimal nutrient availability, feed performance, and overall fish health.

This study is among the earliest to investigate the direct combination of *C. vulgaris* and BSFL as a complete diet for red hybrid tilapia. The promising results pave the way for scaling up this feed formulation and exploring its applicability to other aquaculture species. Further research should focus on optimizing the inclusion levels of *C. vulgaris* and BSFL, evaluating their long-term effects on fish health through assessments of immune response parameters and histological analysis of gut integrity, production, and assessing their potential in integrated multitrophic aquaculture systems. In parallel, incorporating cost–benefit analyses with life cycle assessments would provide a comprehensive understanding of the economic and environmental impacts of this alternative feed. Efforts to standardize the production and processing of *C. vulgaris* and BSFL would also enhance their adoption in the aquafeed industry. Additionally, a detailed amino acid analysis of the experimental diets is essential to accurately compare their profiles with the known nutritional requirements of red hybrid tilapia. Such analyses are critical for optimizing feed formulations, enhancing protein utilization efficiency, and ensuring optimal growth and health outcomes in tilapia aquaculture.

In conclusion, the combination diet achieved comparable growth performance to commercial pellets, with a substantial reduction in feed costs and a notable increase in profitability for fish farmers, reinforcing the practicality of this feed formulation in reducing dependency on conventional feed ingredients like fishmeal and soybean meal. However, the combination diet did not demonstrate significant protection against *S. agalactiae* infection compared to commercial pellets. This highlights the need for further optimization to enhance its immunomodulatory properties. Additionally, the poor growth and survival rates observed in the *C. vulgaris*-only group emphasize the importance of balanced formulations for meeting the nutritional requirements of tilapia. Nevertheless, the findings support the incorporation of *C. vulgaris* and BSFL into aquafeeds, presenting an opportunity for more sustainable and cost-effective tilapia farming while reducing the ecological footprint of aquaculture.

## Figures and Tables

**Table 1:** Cost associated with the experimental diets used in the study.

Group	Details	Price/kg (USD)
G1: Microalgae, *Chlorella vulgaris* biomass	Provided by World New energy Centre Sdn Bhd. with a very minimal cost of production	0.45
G2: Microalgae, *Chlorella vulgaris* biomass + BSFL	Provided by World New Energy Centre Sdn Bhd. with a very minimal cost of production	0.50
G3: Commercial pellet (control)	Star Feed, Klang, Selangor, Malaysia. Size TP1 (1 mm), 32% protein content	1.17

*Note*: The prices for G1 and G2 reflect estimated costs of raw materials only, which are considered the main contributors to the total feed cost, assuming other associated costs are minimal or negligible. In contrast, the price for G3 represents the all-inclusive market price of the final formulated feed product.

**Table 2:** The proximate composition of the experimental diets in the present study.

Composition	*C. vulgaris*-only	BSFL + *C. vulgaris*	Control
^a^Moisture (g/100 g)	7.2	7.3	7.0
^b^Protein (g/100 g)	54.7	34.5	32.2
^c^Total fat, Soxhlet (g/100 g)	0.3	27.0	4.3
^d^Ash (g/100 g)	5.1	7.2	9.6
^e^Total dietary fiber (g/100 g)	16.4	21.9	19.8
^f^Carbohydrate (by different; g/100 g)	32.7	25.0	46.9
^g^Energy (by calculation; kcal/100 g)	352	477	355

^a^In-house Method No. STP/Chem/A04, based on AOAC 20^th^ Edition: 950.46.

^b^In-house Method No. STP/Chem/A03, based on AOAC 20^th^ Edition: 981.10.

^c^In-house Method No. STP/Chem/A02, following AOAC 20^th^ Edition: 991.36.

^d^In-house Method No. STP/Chem/A05, following AOAC 20^th^ Edition: 923.03.

^e^AOAC 20^th^ Edition, 991.43–Enzymatic–Gravimetric Method–MES-TRIS Buffer.

^f^Carbohydrate (by different; g/100 g).

^g^In-house Method No. STP/Chem/A01 following Pearson's The Chemical Analysis of Foods (6^th^ Edition, page 578).

**Table 3:** Growth performance and feed utilization of red hybrid tilapia after a 70-day feeding trial.

Growth parameters	*C. vulgaris*-only	*C. vulgaris* + BSFL	Control
Initial weight (g/fish)	6.3 ± 2.58^a^	6.4 ± 1.96^a^	6.6 ± 2.01^a^
Final weight (g /fish)	8.4 ± 2.88^a^	25.1 ± 9.31^b^	22.7 ± 4.67^b^
Weight gain (g/fish)	2.1 ± 1.34^a^	18.7 ± 1.77^b^	16.1 ± 0.92^b^
Weight gain rate (%)	33.33 ± 4.30^a^	292.19 ± 33.03^b^	243.94 ± 16.13^b^
Specific growth rate (%)	0.41 ± 0.02^a^	1.95 ± 0.18^b^	1.76 ± 0.19^b^
Feed consumption (g)	19.29 ± 0.47^a^	43.01 ± 0.75^b^	38.86 ± 0.12^b^
Feed conversion ratio	9.19 ± 8.45^b^	2.30 ± 0.19^a^	2.41 ± 0.12^a^
Feed efficiency (%)	0.11 ± 0.05^a^	0.43 ± 0.03^b^	0.41 ± 0.04^b^
Survival (%)	7.5 ± 3.54^a^	90 ± 2.83^b^	94.5 ± 0.71^b^

*Note:* Values (mean ± SE) (*n* = 3) in the same row with different superscript lowercase letters show significant differences (*p* < 0.05).

**Table 4:** Economic analysis of red hybrid tilapia-fed on different diets for 70-day feeding trial.

Parameters	*C. vulgaris*-only	*C. vulgaris* + BSFL	Control
Amount of feed used (kg/tank)	1.71	3.87	3.51
Price of feed (USD/kg)	0.45	0.5	1.17
Price of feed/tank	0.009	0.022	0.046
Total fish weight (kg/tank)	0.059	2.033	1.757
Fish price/kg (USD)	2.24	2.24	2.24
Incidence cost (USD)	13.04	0.95	2.34
Incidence cost (% improvement than control)	−457.26	59.40	—
Profit index	0.17	2.35	0.96
Profit index (% improvement than control)	−82.29	144.79	—

## Data Availability

The data supporting this study are available from the corresponding author upon reasonable request.

## References

[1] Suehs B. A., Yamamoto F. Y., Asiri F., Gatlin D. M. (2023). Poly-β-Hydroxybutyrate has Limited Effects on Growth and Immune Responses of Juvenile Hybrid Striped Bass *Morone chrysops* × *M. saxatilis*, and Red Drum *Sciaenops ocellatus* Based on In Vivo and In Vitro Approaches.

[2] Maulu S., Nawanzi K., Abdel-Tawwab M., Khalil H. S. (2021). Fish Nutritional Value as an Approach to Children’s Nutrition. *Frontiers in Nutrition*.

[3] Bartek L., Strid I., Henryson K., Junne S., Rasi S., Eriksson M. (2021). Life Cycle Assessment of Fish Oil Substitute Produced by Microalgae Using Food Waste. *Sustainable Production and Consumption*.

[4] Nagappan S., Das P., AbdulQuadir M. (2021). Potential of Microalgae as a Sustainable Feed Ingredient for Aquaculture. *Journal of Biotechnology*.

[5] Ahmad A., W. Hassan S., Banat F. (2022). An Overview of Microalgae Biomass as a Sustainable Aquaculture Feed Ingredient: Food Security and Circular Economy. *Bioengineered*.

[6] Bora A., Thondi Rajan A. S., Ponnuchamy K., Muthusamy G., Alagarsamy A. (2024). Microalgae to Bioenergy Production: Recent Advances, Influencing Parameters, Utilization of Wastewater—A Critical Review. *Science of the Total Environment*.

[7] Alfiko Y., Xie D., Astuti R. T., Wong J., Wang L. (2022). Insects as a Feed Ingredient for Fish Culture: Status and Trends. *Aquaculture and Fisheries*.

[8] Patyra E., Kwiatek K. (2023). Insect Meals and Insect Antimicrobial Peptides as an Alternative for Antibiotics and Growth Promoters in Livestock Production. *Pathogens*.

[9] Mikołajczak Z., Rawski M., Mazurkiewicz J. (2022). The First Insight into Black Soldier Fly Meal in Brown Trout Nutrition as an Environmentally Sustainable Fish Meal Replacement. *Animal*.

[10] Mohamad S. N., Noordin W. N. M., Ismail N. F., Hamzah A. (2021). Red Hybrid Tilapia (*Oreochromis* spp.) Broodstock Development Programme in Malaysia: Status, Challenges and Prospects for Future Development. *Asian Fisheries Science*.

[11] Huicab-Pech Z., Landeros-Sánchez C., Castañeda-Chávez M., Lango-Reynoso F., López-Collado C., Platas Rosado D. (2016). Current State of Bacteria Pathogenicity and Their Relationship With Host and Environment in Tilapia *Oreochromis niloticus*. *Journal of Aquaculture Research & Development*.

[12] Van Doan H., Lumsangkul C., Sringarm K. (2022). Impacts of Amla (*Phyllanthus emblica*) Fruit Extract on Growth, Skin Mucosal and Serum Immunities, and Disease Resistance of Nile Tilapia (*Oreochromis niloticus*) Raised Under Biofloc System. *Aquaculture Reports*.

[13] Mohd Ali N. S., Saad M. Z., Azmai M. N. A. (2023). Immunogenicity and Efficacy of a Feed-Based Bivalent Vaccine Against Streptococcosis and Motile Aeromonad Septicemia in Red Hybrid Tilapia (*Oreochromis* sp.). *Animals*.

[14] Monir M. S., Yusoff M. S. M., Zulperi Z. M. (2021). Immuno-Protective Efficiency of Feed-Based Whole-Cell Inactivated Bivalent Vaccine Against Streptococcus and Aeromonas Infections in Red Hybrid Tilapia (*Oreochromis niloticus × Oreochromis mossambicus*). *Fish & Shellfish Immunology*.

[15] Limbu S. M., Shoko A. P., Ulotu E. E. (2022). Black Soldier Fly (*Hermetia illucens*, *L*.) Larvae Meal Improves Growth Performance, Feed Efficiency and Economic Returns of Nile Tilapia (*Oreochromis niloticus*, *L*.) Fry. *Aquaculture, Fish and Fisheries*.

[16] Eide L. H., Rocha S. D. C., Morales-Lange B. (2024). Black Soldier Fly Larvae (*Hermetia illucens*) Meal Is a Viable Protein Source for Atlantic Salmon (*Salmo salar*) During a Large-Scale Controlled Field Trial under Commercial-Like Conditions. *Aquaculture*.

[17] Tippayadara N., Dawood M. A. O., Krutmuang P., Hoseinifar S. H., Doan H. Van, Paolucci M. (2021). Replacement of Fish Meal by Black Soldier Fly (*Hermetia illucens*) Larvae Meal: Effects on Growth, Haematology, and Skin Mucus Immunity of Nile Tilapia, *oreochromis niloticus*. *Animals*.

[18] Kals J., Opiyo M. A., Rurangwa E., Soma K., Ndambi A., Vernooij A. (2024). Use of Black Soldier Fly Larvae and Freshwater Shrimp to Partly Substitute Commercial Diet for Nile Tilapia Cultured in Smallholder Fish Farms—A Case Study in Busia County, Kenya. *Frontiers in Sustainable Food Systems*.

[19] Abdel-Tawwab M., Khalil R. H., Abo Selema T. A. M. (2023). Dietary *Chlorella vulgaris* Effectively Alleviates Oxidative Stress, Immunosuppression, and Enhances the Resistance to *Streptococcus agalactiae* Infection in Cadmium-Intoxicated Nile Tilapia Fingerlings. *Fish & Shellfish Immunology*.

[20] Yildirim-Aksoy M., Eljack R., Schrimsher C., Beck B. H. (2020). Use of Dietary Frass from Black Soldier Fly Larvae, *Hermetia illucens*, in Hybrid Tilapia (Nile x Mozambique, *Oreocromis niloticus x O. mozambique*) Diets Improves Growth and Resistance to Bacterial Diseases. *Aquaculture Reports*.

[21] Chen F., Qian J., He Y., Leng Y., Zhou W. (2022). Could *Chlorella pyrenoidosa* be Exploited as an Alternative Nutrition Source in Aquaculture Feed? A Study on the Nutritional Values and Anti-Nutritional Factors. *Frontiers in Nutrition*.

[22] Ma M., Hu Q. (2024). Microalgae as Feed Sources and Feed Additives for Sustainable Aquaculture: Prospects and Challenges. *Reviews in Aquaculture*.

[23] Jiang M., Zhao H. H., Zai S. W., Shepherd B., Wen H., Deng D. F. (2019). A Defatted Microalgae Meal (*Haematococcus pluvialis*) as a Partial Protein Source to Replace Fishmeal for Feeding Juvenile Yellow Perch *Perca flavescens*. *Journal of Applied Phycology*.

